# Isolation of multiple electrocardiogram artifacts using independent vector analysis

**DOI:** 10.7717/peerj-cs.1189

**Published:** 2023-02-09

**Authors:** Zahoor Uddin, Muhammad Altaf, Ayaz Ahmad, Aamir Qamar, Farooq Alam Orakzai

**Affiliations:** Electrical & Computer Engineering, COMSATS Univeristy Islamabad—Wah Campus, Wah Cantt, Punjab, Pakistan

**Keywords:** Electrocardiogram, Artifacts removal, Blind source separation, Independent component analysis, Independent vector analysis

## Abstract

Electrocardiogram (ECG) signals are normally contaminated by various physiological and nonphysiological artifacts. Among these artifacts baseline wandering, electrode movement and muscle artifacts are particularly difficult to remove. Independent component analysis (ICA) is a well-known technique of blind source separation (BSS) and is extensively used in literature for ECG artifact elimination. In this article, the independent vector analysis (IVA) is used for artifact removal in the ECG data. This technique takes advantage of both the canonical correlation analysis (CCA) and the ICA due to the utilization of second-order and high order statistics for un-mixing of the recorded mixed data. The utilization of recorded signals along with their delayed versions makes the IVA-based technique more practical. The proposed technique is evaluated on real and simulated ECG signals and it shows that the proposed technique outperforms the CCA and ICA because it removes the artifacts while altering the ECG signals minimally.

## Introduction

An electrocardiogram (ECG) is an important tool to measure the electrical activity generated by the SA (sinoatrial) node that causes the upper heart chambers (atria) to contract. ECG is an effective tool for investigating the heart related problems like arrhythmia diagnosis and widely adopted in a number of practical applications. ECG signals are utilized for automatic detection of myocardial infarction in Acharya et al. (2017). Kumar, Pachori & Acharya (2017) investigated the ECG signals for detection and characterization of coronary artery disease. Similarly in Acharya et al. (2017), the authors presented the heart failure detection technique based on ECG signals and the extraction of fetal ECG from maternal ECG is achieved in Su & Wu (2017). Qingxue & Zhou (2018) developed person identification technique based on ECG signal processing. Moreover, ECG based silent myocardial infarction as well as long term risk of heart failure is diagnosed in Qureshi et al. (2018). Meanwhile, modern efficient ECG data recording and analysis systems are also been designed even in wireless scenario (Tao et al., 2018; Elgendi et al., 2018; Han et al., 2018; Tanguay et al., 2018; Orphanidou, 2018). However, the recorded ECG signals are normally affected by different types of electro-physiological and non electro-physiological artifacts. The artifacts affected ECG can not be adopted in the sensitive applications. Hence, efficient removal of artifacts is necessary for ECG signals analysis for various applications. Removal of these artifacts before further processing make the design of ECG instrument simpler and produce accurate results.

In literature it is well known that among ECG artifacts, baseline wandering (BW), electrode movement (EM), and muscle artifacts (MAs) are more challenging to separate from the recorded ECG signals (Hesar & Mohebbi, 2017; Varanini et al., 2016; Zarzoso & Nandi, 2001). BW is normally generated through body movements, breathing, and lose sensors contacts. EM is the result of variations of electrodes positions over the human body surface, MA is caused by contraction of the muscles near the electrode (Limaye & Deshmukh, 2016). The main challenges associated with the removal of these artifacts are their unpredictable amplitudes and variable frequency range (Hegde, Deekshit & Satyanarayana, 2012).

### Related work

Numerous researches have contributed to artifacts removal from ECG signals, using algorithms like, extended Kalman filter (Hesar & Mohebbi, 2017), least mean square (LMS) (Rahman, Shaik & Reddy, 2009) and Weiner filter (Chang & Liu, 2011), *etc*. ECG signal de-noising and classification schemes based on projected and dynamic features are presented in Chen et al. (2017). High density muscle noise removal from the recorded ECG signal is performed in Wang et al. (2020) using the independent vector analysis (IVA) technique. Separation of the fetal and maternal ECG signals is carried out in Sugumar & Vanathi (2016) through the IVA technique. Successive local filtering based denoising is discussed in Mourad (2022). Deep learning based ECG de-noising technique is proposed in Rahhal et al. (2016) and Rasti-Meymandi & Ghaffari (2022). The segmented beat classification and de-noising method discussed in Agostinelli et al. (2016), proposed a filtering technique to suppress the noise followed by the detection of QRS complex from the ECG signals using the MIT-BIH Noise Stress Test Database. Time-series clustering techniques used for ECG classification and artifacts removal in Rodrigues, Belo & Gamboa (2017), extract the best characterize features of the signal over time and group its samples in individual clusters through an agglomerative clustering approach. Moreover, the blind source separation (BSS) technique called the independent component analysis (ICA) is also used for fetal ECG extraction and artifacts removal in Varanini et al. (2016), Sameni et al. (2007), and Jafari & Chambers (2005). ECG signal classification and de-noising are also performed in Uddin & Alam (2009), Sameni, Jutten & Shamsollahi (2008), Vayá et al. (2007), and Rieta et al. (2004) using ICA. In all these applications mixed data is first recorded through electrodes and then processed using ICA algorithms for un-mixing and further classifications. The IVA technique is already used for gradient noise removal from electroencephalogram signals in Acharjee et al. (2015).

Adaptive filtering techniques and ICA are used for ECG artifacts removal; however, in Zarzoso & Nandi (2001), it is shown that ICA outperforms the adaptive filtering techniques for ECG artifacts removal. Moreover, ICA is recommended by various researchers for artifacts removal but some inefficiency of ICA is also reported in Urrestarazu et al. (2004) and Shackman et al. (2009). In literature, canonical correlation analysis (CCA) is used as an alternative to ICA (De Clercq et al., 2006), which is yet not used for ECG artifacts removal. CCA utilizes the original signals as well as the delayed versions of the signals. It is based on second-order statistics (SOS) and extracts maximally auto-correlated and mutually un-correlated signals (De Clercq et al., 2006). From Mowla et al. (2015), it is known that CCA is an efficient and practically useable technique as compared to ICA. Moreover, ICA utilizes high order statistics (HOS) to explore statistical independence while CCA is based on SOS to recover statistically un-correlated sources. It is clear from the statistical theory that un-correlatedness is a weaker condition than independence.

A recently developed technique of BSS called the independent vector analysis (IVA) combines the advantages of both ICA and CCA in a single framework (Anderson et al., 2014). IVA processes the original and time-delayed versions of the signals (just like CCA) while utilizing the HOS (like ICA). IVA assumes that the source signals in one data set are independent of each other and at least one source is dependent on one source of the other data set. Moreover, from Anderson et al. (2014) it is known that IVA performs well as compared to ICA and CCA.

### Contribution

It is clear from the literature that the ICA algorithms perform well as compare to adaptive filtering techniques like weiner filter, kalman filter *etc*. as shown in Mohammed, Hassan & Ferikoglu (2021), Maghrebi & Prouff (2018), Martinek et al. (2021), Maghrebi & Prouff (2018), Uddin et al. (2020), and Villena et al. (2018). Mohammed, Hassan & Ferikoglu (2021) in particular mentioned that the ICA algorithm gives more accurate results than the extended kalman filter in reducing baseline wandering and electrode movement artifacts. It is also important to mention that in case of low frequency applications ICA gives more accurate results (Mohammed, Hassan & Ferikoglu, 2021; Villena et al., 2018).

Based on this discussion, an IVA based technique is proposed in this article for ECG artifacts removal. This is the first article that proposes the IVA-based technique for ECG artifacts removal. The IVA-based technique produces more clear and visible ECG signals that might help medical specialists to observe some very low amplitude electro-physiological effects of the heart. In this article, the performance of the three IVA algorithms called the IVA-L, the IVA-G, and the IVA-GGD is investigated for ECG artifacts removal. The IVA-L algorithm utilize the HOS and assumes Laplacian distribution for the source component vectors (Kim et al., 2007). The IVA-G algorithm exploits linear dependencies without taking into account the HOS. The IVA-G algorithm assumes Gaussian distribution for the mixing sources (Anderson, Adali & Li, 2012). The IVA-GGD algorithm utilizes both the SOS and HOS while assuming multivariate generalized Gaussian distribution for the underlying sources (Anderson et al., 2014). It is also important to mention that all the data is taken from the MIT-BIH Noise Stress Test Database for ECG and artifacts signals (Moody, Muldrow & Mark, 1984). The MIT-BIH Noise Stress Test Database is freely available for further research on ECG signal processing. In addition, the main contributions of this research are as follows:

- Recently developed BSS technique, the IVA is used to separate artifacts from ECG signal.
- The three most challenging ECG artifacts BW, MA and the EM are considered to remove from the recorded ECG signals.
- Performance of the ICA, CCA and IVA are analyzed for artifacts removal utilizing real simulated ECG signals.
- Three variants of IVA, the IVA-L, IVA-G, and IVA-GGD are investigated to study their performance for ECG artifacts removal.

The rest of the article is organized such that Section 2 presents details of the ECG data. Both the realistic simulated and real ECG signals along with ECG artifacts are discussed. The system model is given in Section 3, while the proposed algorithm using IVA algorithms is discussed in Section 4. A simulation study of the simulated and real signals is carried out in Section 5 with the concluding remarks in Section 6.

*Notations*: Lowercase letters are used for scalars (*e.g*., $x$, $y$, $z$,…), lowercase boldface letters for vectors (*e.g*., ${\textbf {x}}$, ${\textbf {y}}$, ${\textbf {z}}$,…), and uppercase boldface letters for matrices (*e.g*., ${\textbf {X}}$, $\textbf {Y}$, $\textbf {Z}$,…). Transpose is denoted by uppercase superscript *T* (*e.g*., $\textbf {x}^{T}$, $\textbf {X}^{T}$).

## Ecg data and artifacts

Realistic simulated and real ECG data are considered for simulations. Real data is taken from the MIT-BIH database (Moody, Muldrow & Mark, 1984). The acquired signals in the MIT-BIH Noise Stress Test Database are digitized using uni-polar ADCs with 11-bit resolution. This database is open source for further research. The MIT-BIH database contains the ECG signals and their artifacts. The artifacts considered in this work are as follows:

- **Muscle artifact**: Muscle artifacts are the results of muscle contraction having low amplitudes and a large frequency range from 0–10 kHz.
- **Baseline wandering**: Baseline wandering originates due to body movements, breathing, and loose sensor contact. Body movements cause unpredictable large amplitude and low-frequency artifacts. Breathing also causes low frequency drifting between 0.15 and 0.3 Hz.
- **Electrode movement**: Electrode movement is generated due to electrode position away from the skin contact, changing the electrode and skin impedance causing potential variations in the recorded ECG signal.
- **Other artifacts**: Other ECG artifacts include power line interference, device noise, Electro-surgical noise, quantization noise, aliasing, *etc*.

**Figure 1:**
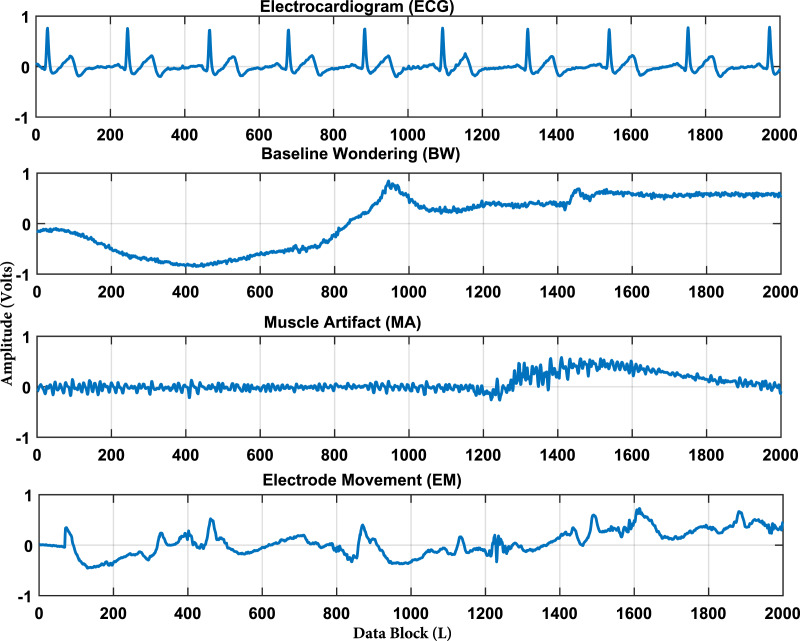
ECG and artifacts signals from MIT-BIH noise stress test database. The artifacts signals contains baseline wondering (BW), muscle artifacts (MA), and the electrode movement (EM) artifacts. It is also important to note that the x-axis represents the number of sample and the y-axis shows the amplitude of the recorded signal.

The time-domain real ECG, BW, EM, and MA signals are demonstrated in Fig. 1 with 2,000 data samples of each signal as a first data set.

**Figure 2:**
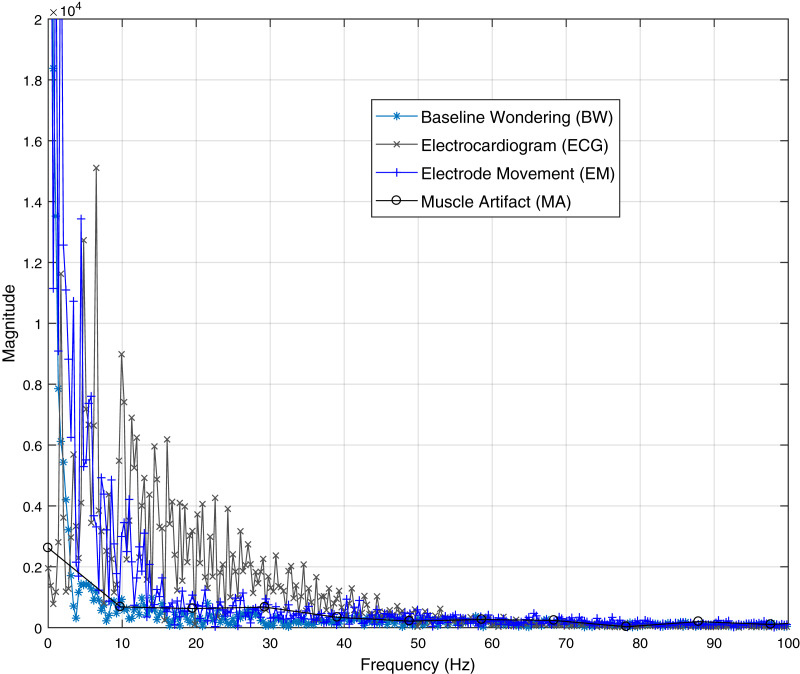
Frequency domain ECG and artifacts signals. This figure will show clearly the overlapped region of the ECG and artifacts signals. We demonstrate the low frequency part where most of the frequencies overlapped with high amplitude.

**Figure 3:**
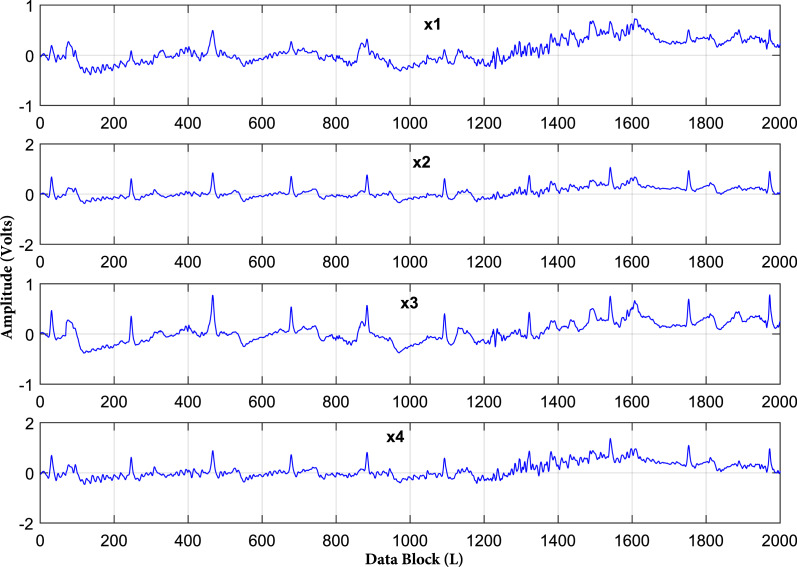
Recorded ECG signals in the presence of artifacts for a single data set at SNR of 20 dB. The recorded mixed ECG signals are shown for a single data set with data block length L = 2,000 samples.

Frequency domain representation is shown in Fig. 2. It shows that most of the frequencies of ECG and artifacts lie in the range of 50 Hz. From Fig. 2 it is observed that all the frequencies of ECG signal and artifacts overlap with each other. Hence, to cleanly extract these ECG signals, some efficient BSS techniques are required. As it is already discussed, IVA is the more efficient BSS technique as compared to ICA and CCA (Moody, Muldrow & Mark, 1984). Based on the discussions, it is recommended to utilize IVA for ECG signals de-noising. Moreover, the recorded mixed ECG data is shown in Fig. 3 for a single data set with *L* = 2,000 samples. The measurement is taken in the presence of additive white Gaussian noise (AWGN) with signal to noise ratio (SNR) of 20 dB. Figure 3 basically contains mixture signals of all the individual source signals. The source signals are ECG, BW, EM and MA. The mixing process is performed in MATLAB such that the source signals matrix $ {S}$ of size $4\times 2,\!000$ is multiplied with a randomly generated mixing matrix $ {A}$ of size $4\times 4$. Mixed data is recorded in matrix $ {X}$, where $ {X}$ is of size $4\times 2,\!000$. It must be noted that in case of ICA a single data set as shown above is utilized while in case of IVA multiple copies of the source signals are recorded and processed for un-mixing *i.e*., multiple mixing matrices are observed and un-mixing is performed at a time. This is the main advantage of the IVA algorithm to un-mix the recorded signals and its delayed versions at a time. The realistic simulated ECG signals are generated in MATLAB version R2016a (MathWorks, Inc., Natick, MA, USA).

## System model

This section presents the ECG signals and artifacts in the IVA data model. *K* number of independent sources *i.e*., ECG and artifacts are considered and all sources contain *L* number of samples for *D* data sets. The acquired data using ECG electrodes is expressed as:

(1) $${X}^{d}=\textbf{A}^{d}\textbf{S}^{d} \quad 1\leq d \leq D,$$

The matrices $\textbf {S}^{d}$ contains the source data vectors $\textbf {s}{^{d}}_{1}, \textbf {s}{^{d}}_{2}{,...,}\textbf {s}{^{d}}_{K}$, where every vector having length *L*. All vectors are real valued random vectors having zero mean. The mixing matices $\textbf {A}^{d}$ are also real with random values for *D* number of data sets. Hence, the the IVA algorithm responsible to estimate these unknown matrices while utilizing the mixed data. The source data is represented by $(\textbf {S}^{1})^{T}, (\textbf {S}^{2})^{T},...,(\textbf {S}^{D})^{T}$ in *D* data sets. After the estimation of $\textbf {A}^{d}$ through the IVA algorithm, the resultant source signals as given in Qamar et al. (2022) are expressed as:

(2) $${Y}^{d}=\textbf{W}^{d}\textbf{X}^{d}$$

The $\textbf {W}^{d}$ is inverse of $\textbf {A}^{d}$ and is called the un-mixing matrix estimated for *D* data sets. The estimated source data vectors are $\textbf {y}_{1}^{d}, \textbf {y}_{2}^{d},...,y_{k}^{d}.$

## Proposed iva-based ecg artifacts separation

Multi-channel ECG signals are recorded in the presence of various artifacts *i.e*., BW, EM, and MA as well as noise. The number of ECG and artifact signals are denoted by *K*, each signal has data block length *L* with *D* number of data sets. The recorded mixed data contains *D* number of data sets $(\textbf {X}^{1})^{T}, (\textbf {X}^{2})^{T},...,(\textbf {X}^{D})^{T}$ as shown in Fig. 3 for SNR of 20 dB. Since, the artifact signals have overlapped frequencies with the original ECG signal as illustrated in Fig. 2, the role of the BSS algorithms is to estimate the source signals from the recorded mixed signals. The BSS algorithms know nothing except independence and non-Gaussianity of the source signals.

The estimated sources of each BSS algorithm have scaling and order ambiguities. The scaling issue can be easily resolved considering the source signals with unit variance and also scaling the un-mixing vector to extract the unit variance sources. The arbitrary order of the estimated signals in each data set can be corrected using the permutation matrix, which is common in each data set (Anderson, Adali & Li, 2012).

The IVA algorithms separate the mixed recorded signals as a first data set and their delayed versions as other data sets. This separation is performed using the minimization of the mutual information among the estimated source component vectors (SCVs). The cost function of IVA is demonstrated in Anderson, Adali & Li (2012) and illustrated here as:

(3) $${I_{IVA}} = \sum\limits_{k = 1}^K {\left( {\sum\limits_{d = 1}^D H [y_k^d] - I[{y_k}]} \right)}  - \sum\limits_{d = 1}^D {\log |{W^d}| - C} $$

The $I[y_k]$ represents mutual information within $k_{th}$ SCVs. *H* is the entropy, ${\textbf {W}^d}$ is the un-mixing matrix of $d_{th}$ data set and *C* is a constant factor which is equivalent to $H[\textbf {X}^1,\textbf {X}^2,...,\textbf {X}^D]$ depending only on the recorded mixed data. The IVA algorithms minimize the cost function of Acharya et al. (2017) and maximizes the mutual information within each SCV.

ICA is a well-known blind source separation technique used for linearly mixed signals utilizing statistical independence of the source signals (Uddin et al., 2015). CCA considers the mixed recorded signals as well as its delayed versions by exploiting the SOS. The IVA combines the advantages of CCA and ICA by exploiting the SOS and HOS. Moreover, numerous variants of IVA algorithms, such as IVA-GGD (Anderson et al., 2014), IVA-L (Kim et al., 2007) and IVA-G (Anderson, Adali & Li, 2012) exist in literature and their dominance is already proven. Motivated by this, this research implemented various versions of IVA algorithms to verify their validity for ECG artifacts removal. All these algorithms utilize the IVA cost function given in Acharya et al. (2017) to estimate the un-mixing matrices. In the case of complex-valued data, the IVA-G algorithm includes the pseudo-co-variance matrix in the cost function. This algorithm also ignores the HOS and sample to sample dependency. The IVA-L utilizes the HOS for un-mixing while ignoring the sample to sample dependency and SOS. The matrix gradient approach is used in the implementation of the IVA-L algorithm. The IVA-GGD algorithm utilizes the HOS and SOS for source signal estimation considering multivariate Gaussian prior. This algorithm also avoids sample to sample dependency. Moreover, processing of the original as well as the delayed versions makes the IVA algorithms more practical compared to the ICA technique. Based on these advantages, various variants of IVA algorithms are implemented in this article and their performance is tested for the ECG artifacts removal.

## Simulation results

In this section, simulation results of the proposed IVA based technique for ECG artifacts removal from the recorded mixed signals is presented. The IVA algorithms considered for simulations are IVA-GGD, IVA-L and IVA-G. Performance of these algorithms is evaluated for various SNRs ranging from 0 to 20 dB. Results are compiled using Monte Carlo simulation. The ECG artifacts considered for simulation are baseline wandering (BW), electrode movement (EM), and mascle artifacts (MA). Real and simulated ECG signals are utilized in the simulations. The real ECG signals are downloaded from MIT-BIH database and the simulated ECG signals are generated in MATLAB. The number of source signals considered are *K* = 4, the number of data sets *D* = 4, and length *L* of the processing data blocks in each data set ranges from 50 to 2,000 samples. Moreover, to evaluate the effectiveness of the proposed IVA technique for ECG artifacts removal different performance evaluation criterion are used that are given below:

- The corresponding root mean square error $(CRMSE)$ used in Chen et al. (2017) is expressed below:

(4) $$CRMSE = {{RMS({\bf{s}}_{ECG}^d - {\bf{y}}_{ECG}^d)} \over {RMS({\bf{s}}_{ECG}^d)}}$$

- The $\textbf {s}^{d}_{ECG}$ and $\textbf {y}^{d}_{ECG}$ represent the original simulated ECG and the reconstructed ECG signals simultaneously at data set $d$.
- Common inter-symbol-interference ( $ISI_{com}$) (Anderson, Adali & Li, 2012) is also utilized as a performance measure that is presented as:

(5) $$ISI_{com}={\frac{1}{2K(K-1)}}\left[{\psi^{\prime}+\psi^{\prime\prime}}\right]$$

- The ${\psi ^\prime } = \sum _{n = 1}^K\left( {\sum _{m = 1}^K{{g_{m,n}^\prime } \over {ma{x_p}g_{n,p}^\prime }} - 1} \right)$
${\psi ^\prime }^\prime  = \sum _{m = 1}^K\left( {\sum _{n = 1}^K{{g_{m,n}^\prime } \over {ma{x_p}g_{n,p}^\prime }} - 1} \right)$
  and $\textbf {G}^d=\textbf {W}^{d}\textbf {A}^{d}$ with $g_{m,n}=\sum _{d=1}^{D}|g_{m,n}^{d}|$. The $ISI_{com}$ is normalized so that its maximum value is one and minimum vale is zero, where zero value corresponds to ideal separation performance.
- The $U_{W^{d}A^{d}}$ is utilized as another evaluation criteria and is expressed as:

(6) $$U_{W^{d}A^{d}}={\sum_{n=1}^{K}\left(\sum_{m=1}^{K} {g^{\prime}_{m,n}}-1\right)}$$

The ideal separation corresponds to zero value of $U_{W^{d}A^{d}}$.

**Figure 4:**
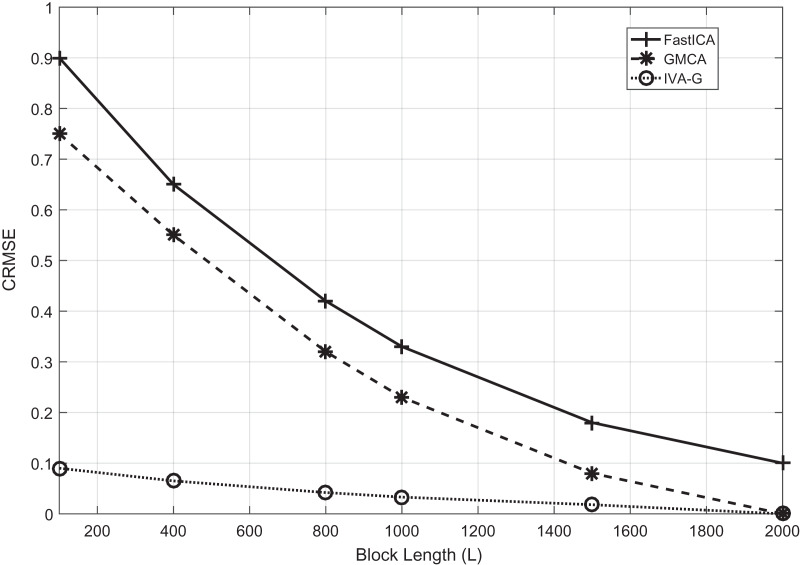
The *CRMSE* performance comparison of the Fast-ICA, GMCA and IVA-G algorithms for ECG artifacts removal. Performance evaluation is carried out for different values of L ranging from 100 to 2,000 samples in a single data set.

**Figure 5:**
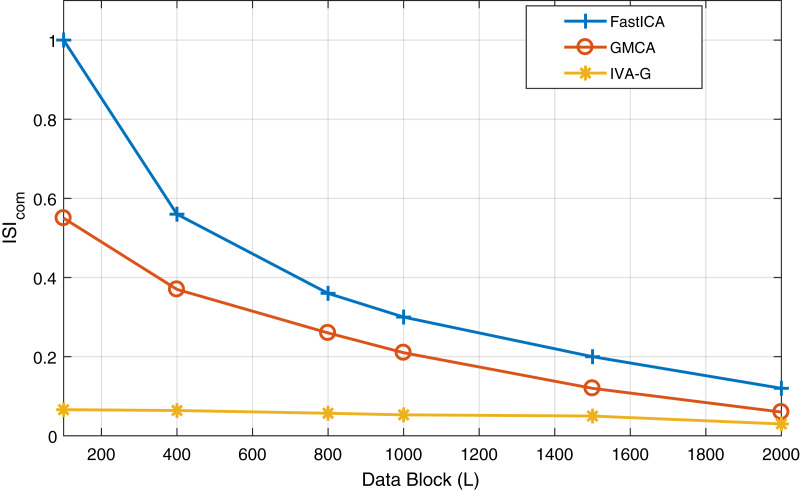
The $ISI_{com}$ performance of all the three algorithms *i.e*., Fast-ICA, GMCA and IVA-G algorithms for ECG artifacts removal. Performance evaluation is carried out for different values of L ranging from 100 to 2,000 samples in a single data set at SNR of 20 dB.

**Figure 6:**
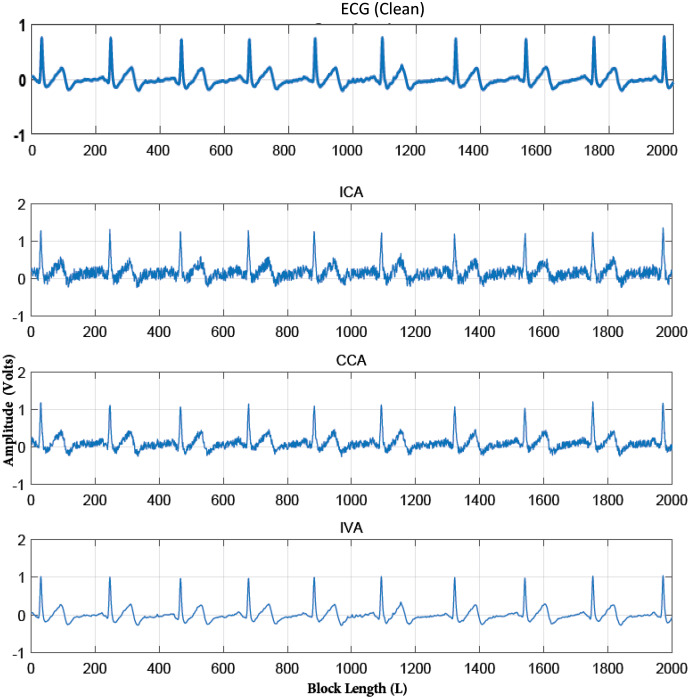
Extracted and actual ECG signals of Fig. 4 for all the three algorithms (FastICA, GMCA, IVA-G) to observe the effect of AWGN noise over these algorithms.

First, the effectiveness of the IVA-based technique in comparison with ICA and CCA techniques is demonstrated. The results of the three techniques are demonstrated while utilizing the Fast-ICA algorithm (Uddin, Ahmad & Iqbal, 2017) of the ICA, the GMCA algorithm (Li et al., 2009) of CCA and the IVA-G algorithm of the IVA. Simulations are performed at an SNR of 20 dB. The performance evaluation criteria used is *CRMSE*. In the case of the ICA algorithm, the value of the data set is one. Performance evaluation is carried out for different values of *L* ranging between 100 to 2,000 samples in a single data set. The results of ICA, CCA and IVA algorithms are demonstrated in Fig. 4. The simulation results clearly show that the IVA outperforms ICA and CCA algorithms. These results also verify that the IVA algorithm is less sensitive to the processing data block lengths. The performance improvement at a block length of $L=100$ is around 85 $\%$ for the IVA technique and 15 $\%$ for the CCA technique as compared with the ICA technique. Similarly, we demonstrate the $ISI_{com}$ performance of all these algorithms for the same conditions as given in the above simulations. The results are demonstrated in Fig. 5. This figure also shows the effective performance of the IVA algorithm. The extracted ECG signals for these three algorithms are also demonstrated in Fig. 6. It shows that the IVA algorithm outperforms other algorithms and is also less sensitive to AWGN noise.

**Figure 7:**
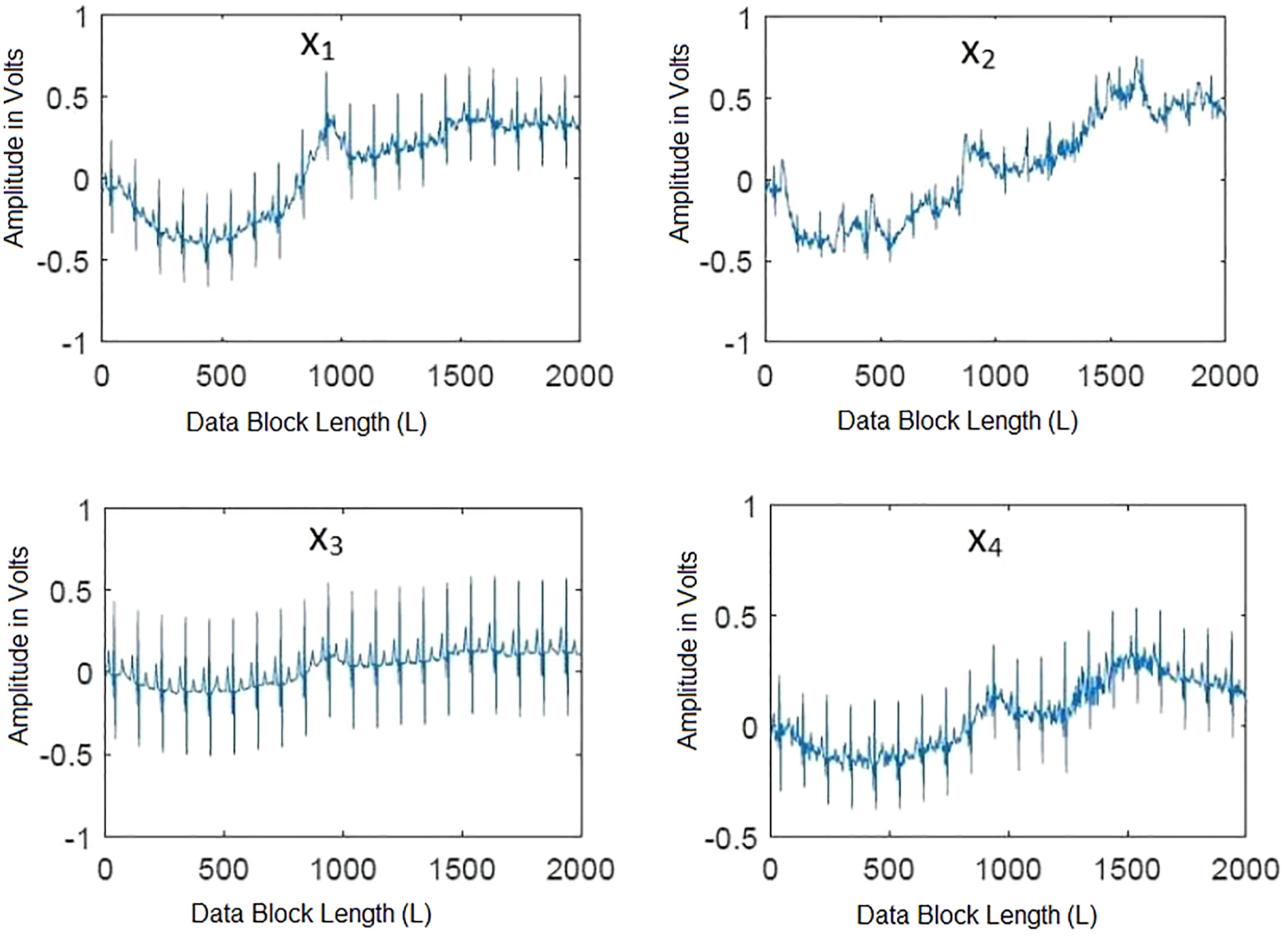
Mixtures of the simulated ECG and artifacts signals at SNR = 20 dB. Linearly mixed instantaneous signals are generated using randomly generated mixing matrices in MATLAB.

Second, the quality of the separated ECG signals from various artifacts using the IVA algorithms using ( $ISI_{com}$) is evaluated. Here, the simulated ECG signal corrupted by various artifacts *i.e*., BW, MA, and EM is considered. Linearly mixed instantaneous signals are generated using randomly generated mixing matrices in MATLAB. The mixed recorded signals are shown in Fig. 7 for a single data set. The mixing process of Figs. 3 and 7 is same, the difference in signals is such that Fig. 3 contains the simulated ECG signals and Fig. 6 shows the realistic ECG signal. Three IVA algorithms are applied to the simulated ECG signals for artifacts removal. The reliability of the ECG signals for all three algorithms is evaluated for different values of SNRs. The simulations are performed over four recorded data sets independently. In each run, the pure ECG signal is extracted and artifacts are separated from the recorded mixed signals.

**Figure 8:**
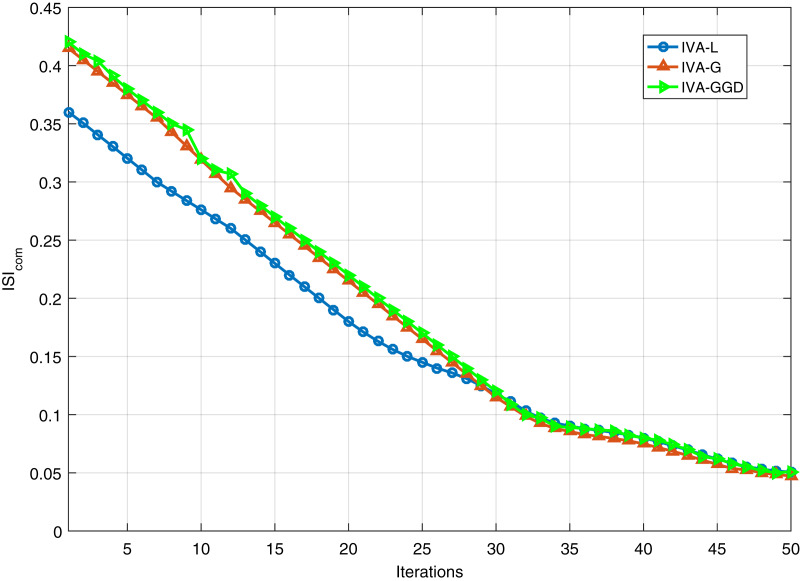
$ISI_{com}$ performance of the IVA-GGD, IVA-G and IVA-L algorithms at SNR of 20 dB.

**Figure 9:**
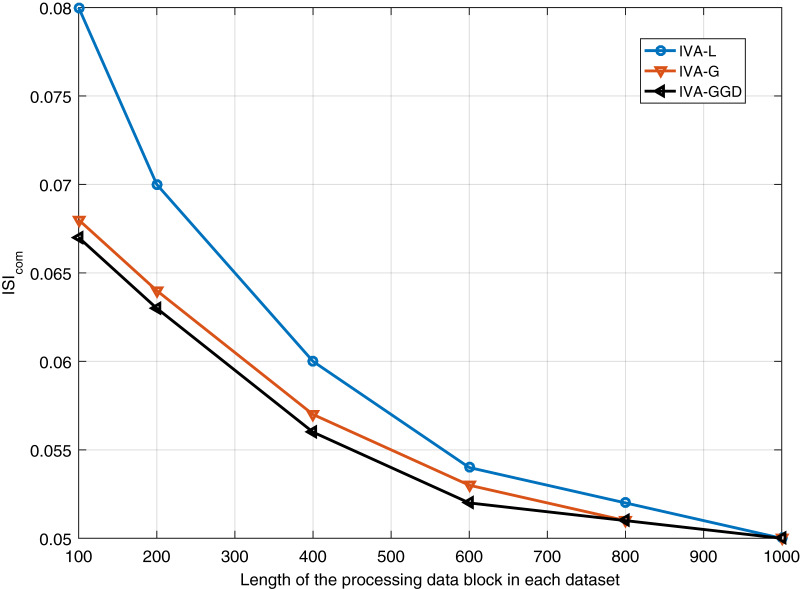
Results of all the three IVA algorithms for different values of the input data block lengths in different data sets.

The $ISI_{com}$ performance of the IVA algorithms for different number of iterations is performed. Results are shown in Fig. 8 for 20 dB SNR and a block length of 1,000 samples. It shows similar performance of all the algorithms at steady state condition. Furthermore, performance of the IVA algorithms is also evaluated for different values of the input data block lengths in different data sets. Simulation results are shown in Fig. 9 at 20 dB SNR. These results show that the IVA-L algorithm is more sensitive to length of the processing data blocks. At a block length of 100 samples in each data set the performance improvements of the IVA-G and IVA-GGD are $18\%$ and $19\%$ as compared to the IVA-L.

**Figure 10:**
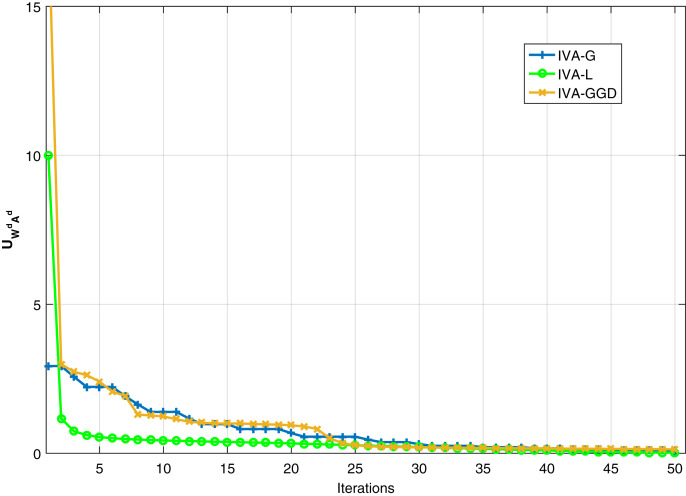
Convergence behavior of the IVA-GGD, IVA-G and IVA-L algorithms at 20 dB SNR.

In order to further investigate the IVA algorithms, we evaluate the $U_{W^{d}A^{d}}$ performance of the IVA algorithms at SNR of 20 dB for different number of iterations. Results are given in Fig. 10. It shows that the IVA-L converges faster as compared to IVA-G and IVA-GGD algorithms. The IVA-L converges at approximately 10 iterations, the other two converges at 25 iterations approximately. Although the IVA-L converges fast with same steady state results as achieved by other algorithms.

**Figure 11:**
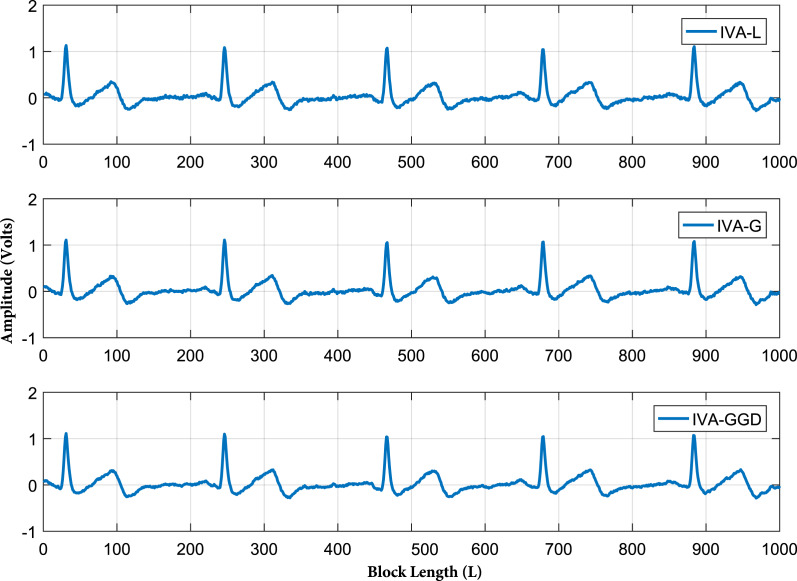
Extracted ECG signals of all the three IVA algorithm. The IVA algorithms considered are IVA-L, IVA-G and IVA-GGD.

**Figure 12:**
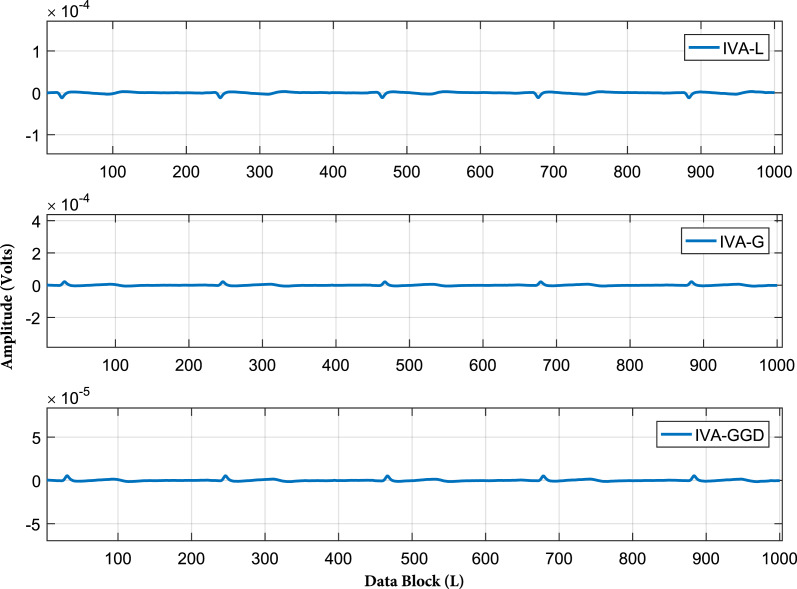
Error signals of all the three IVA algorithms at SNR of 20 dB. The data block length utilized is 1,000 samples in each data set, where error signal is the difference of the real and separated ECG signals. The resultant very low amplitudes of the error signals shows effectiveness of the IVA algorithms.

In the third part of simulations, we demonstrate the practical performance of the IVA algorithms for real ECG artifacts removal. The ECG artifacts considered in this part are BW, EM and MA. Removal of these artifacts is a challenging task due to their variable amplitudes and frequencies. The IVA algorithms considered in this section are IVA-L, IVA-G and IVA-GGD. The separated signals of the IVA algorithms are shown in Fig. 11 for 20 dB SNR. The results shows that three algorithms perform well for ECG artifacts removal. Moreover, the error signals are also demonstrated in Fig. 12, where error signal is the difference of the real and separated ECG signals. The resultant very low amplitudes of the error signals shows the effectiveness of the IVA algorithms.

The algorithms performance is also investigated for different values of the input data block lengths in each data sets. The $ISI_{com}$ performance is evaluated and the results are shown in Table 1. The data block lengths considered in each data set ranges from 50 to 2,000 samples with SNR of 20 dB. These results show that the IVA-GGD and IVA-G are less sensitive to lengths of the processing data blocks as compared to IVA-L. Furthermore, the algorithms performance is also evaluated for various SNR values with input data block length of 2,000 samples in each data set. The results are demonstrated in Table 2 for SNR ranges from 0 to 20 dB. Performance of the algorithms degrade for lower values of SNR. The IVA-GGD and IVA-G provide a little better results as compared to IVA-L for lower SNR values.

**Table 1:** The $ISI_{com}$ performance of the real ECG for all the three IVA algorithms *i.e*., IVA-GGD, IVA-L, and IVA-G at SNR of 20 dB. The algorithms performance is evaluated for different values of the input data block lengths ranges from 50 to 2,000 samples in each data sets.

L	IVA-L	IVA-G	IVA-GGD
50	0.10	0.058	0.0570
100	0.057	0.052	0.051
500	0.053	0.051	0.0507
1,000	0.05	0.05	0.05
2,000	0.05	0.05	0.05

**Table 2:** The $ISI_{com}$ results of the real ECG for all the three IVA algorithms at input data block length of 2,000 samples in each data set and different SNRs that ranges from 0 to 20 dB.

SNR in dB	IVA-L	IVA-G	IVA-GGD
0	0.923	0.655	0.644
5	0.433	0.203	0.2007
10	0.157	0.141	0.140
20	0.0501	0.05	0.05

**Figure 13:**
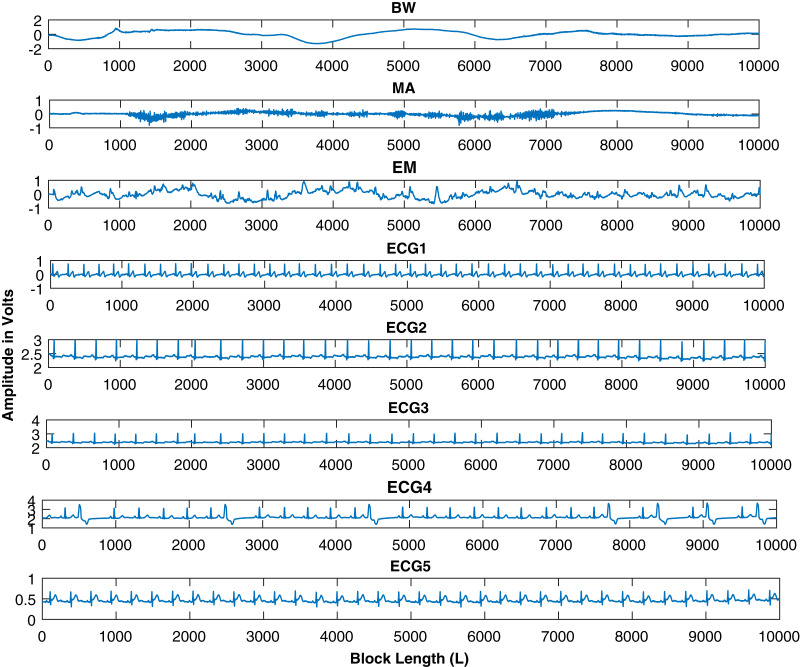
Source signals of ECG, BW, MA, and EM, downloaded from the MIT-BIH database. Five ECG signals are shown for further processing with data block lengths of 10,000 samples.

**Figure 14:**
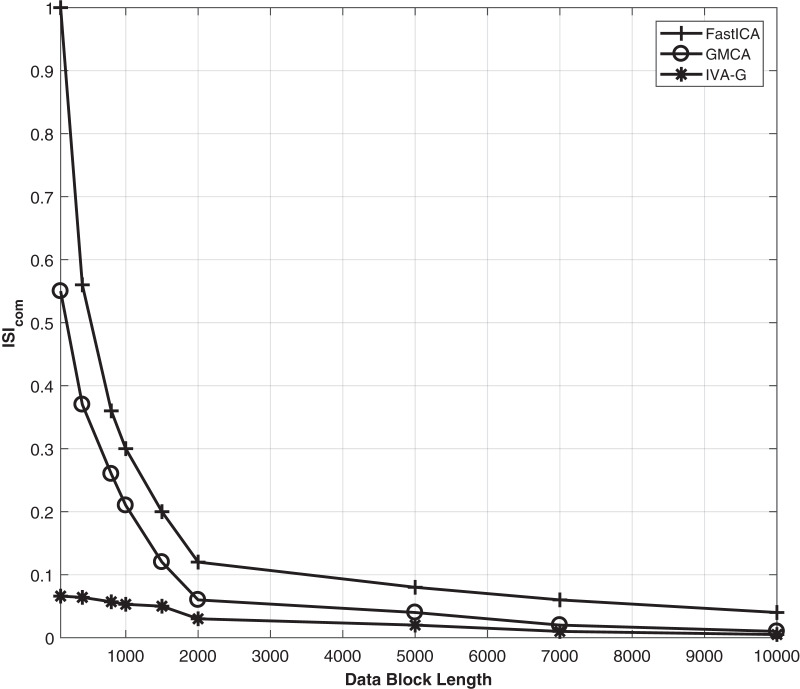
Results of the IVA-G, GMCA, and FastICA algorithms for different values of the input data block lengths range from 100 to 10,000 samples in different data sets. *ECG_2_* is utilized in these simulations.

**Figure 15:**
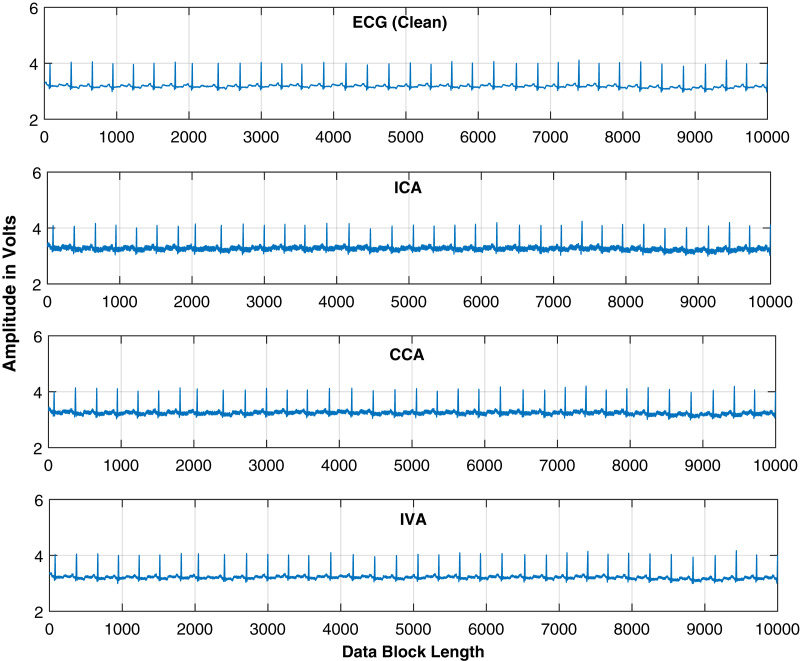
Extracted ECG signals are shown for the IVA-G, GMCA, and FastICA algorithms while utilizing block length of 10,000 samples. ECG signal utilized is *ECG_2_*.

Finally, larger data blocks with more ECG signals are considered. ECG and interfering source signals utilized in this part of simulations have data block lengths ranging from 100 to 10,000 samples. Five ECG signals *i.e*., *ECG_1_*, *ECG_2_*, *ECG_3_*, *ECG_4_* and *ECG_5_* are considered from the MIT-BIH database for further analysis. The source signals are shown in Fig. 13. This figure contains the BW, EM, MA and ECG signals. Larger samples are considered to further investigate the behavior of the ICA, CCA, and IVA algorithms. The mixing and un-mixing procedures are performed as discussed above. The *ISI_com_* performance of all three algorithms is evaluated while considering the 20 dB SNR. Results of all the five ECG signals *i.e*., *ECG_1_*, *ECG_2_*, *ECG_3_*, *ECG_4_* and *ECG_5_* are demonstrated in Table 3. This table shows approximately the same performance of a single algorithm for all five ECG signals. The *ISI_com_* performance of the *ECG2* is also demonstrated in Fig. 14 to observe the performance improvement for increased lengths of the processing data blocks. Although, in addition to Table 3 the results of all the other ECG signals can also be included as figures but restricted to *ECG2* only to avoid the unnecessary length of the article. Furthermore, the reconstructed ECG signal *i.e*., *ECG2* in Fig. 15 is also demonstrated for all three algorithms.

**Table 3:** The ISI_*com*_ results of the IVA-G, GMCA, and FastICA algorithms are demonstrated for different values of the input data block lengths ranges from 100 to 10,000 samples in different data sets. All the five ECG signals are considered in these simulations while utilizing 20 dB SNR.

L	100	400	800	1,000	1,500	2,000	5,000	7,000	10,000
IVA-G*_ECG1_*	0.056	0.054	0.051	0.0503	0.0490	0.0401	0.02	0.01	0.00504
IVA-G*_ECG2_*	0.0554	0.0536	0.052	0.05026	0.0481	0.041	0.021	0.011	0.005
IVA-G*_ECG3_*	0.0557	0.0541	0.0508	0.05029	0.04914	0.04012	0.0201	0.0102	0.0049
IVA-G*_ECG4_*	0.055	0.054	0.0509	0.0502	0.0479	0.04010	0.0202	0.0102	0.0050
IVA-G*_ECG5_*	0.0561	0.0639	0.052	0.05028	0.0490	0.0402	0.0203	0.0103	0.0051
GMCA*_ECG1_*	0.55	0.37	0.26	0.21	0.12	0.06	0.04	0.02	0.01
GMCA*_ECG2_*	0.551	0.3701	0.2602	0.209	0.1204	0.0612	0.0411	0.021	0.0109
GMCA*_ECG3_*	0.5502	0.371	0.261	0.212	0.1201	0.0610	0.041	0.0206	0.0108
GMCA*_ECG4_*	0.550	0.37	0.2601	0.2101	0.1202	0.0613	0.041	0.0208	0.0109
GMCA*_ECG5_*	0.5502	0.371	0.261	0.212	0.1201	0.0612	0.0411	0.0209	0.0107
FastICA*_ECG1_*	0.99	0.561	0.361	0.303	0.2	0.1202	0.08	0.06	0.041
FastICA*_ECG2_*	0.998	0.56	0.36	0.3	0.201	0.1203	0.0802	0.061	0.0404
FastICA*_ECG3_*	0.997	0.5601	0.362	0.31	0.21	0.12	0.081	0.0601	0.04
FastICA*_ECG4_*	0.997	0.561	0.361	0.309	0.201	0.120	0.0811	0.0611	0.0401
FastICA*_ECG5_*	0.9909	0.5603	0.3606	0.3108	0.210	0.1204	0.0810	0.06009	0.0402

## Discussion and conclusion

The ECG artifacts removal problem is investigated in this article. Both realistic simulated and real ECG signals are utilized for simulation. The artifacts considered are baseline wandering, electrode movement and muscle artifacts. Removal of these artifacts is difficult due to their variable amplitudes and frequencies. The IVA technique is compared in this article shows that it outperforms the CCA and ICA techniques. We further investigated the IVA technique for ECG artifacts removal. For comparison purpose, we consider three IVA algorithms to get more clear ECG signals in the presence of various artifacts. In addition, we utilized different evaluation criterion to confirm performance of the proposed technique. The $ISI_{com}$ performance of the IVA algorithms for different values of the input data block lengths in different data sets. Simulation results are shown in Fig. 9 at 20 dB SNR. These results show that the IVA-L algorithm is more sensitive to length of the processing data blocks. At a block length of 100 samples in each data set the performance improvements of the IVA-G and IVA-GGD are $18\%$ and $19\%$ as compared to the IVA-L. As a concluding remarks, we can say that the IVA algorithms are less sensitive to input data block lengths and input SNRs as compared to the ICA technique. Thus, IVA is proved to be an efficient and more practical technique for ECG de-noising.

## Supplemental Information

10.7717/peerj-cs.1189/supp-1 Supplemental Information 1 Raw data.

10.7717/peerj-cs.1189/supp-2 Supplemental Information 2 Code.

## Additional Information and Declarations

### Data Availability

The following information was supplied regarding data availability: The data is available at the MIT-BIH Arrhythmia Database Directory: Moody GB, Muldrow WE, Mark RG. A noise stress test for arrhythmia detectors. Computers in Cardiology 1984; 11:381–384. https://archive.physionet.org/physiobank/database/html/mitdbdir/mitdbdir.htm. The raw data and codes are available in the Supplemental Files.
